# As good as physicians: patient perceptions of physicians and non-physician clinicians in rural primary health centers in India

**DOI:** 10.9745/GHSP-D-13-00085

**Published:** 2013-10-07

**Authors:** Krishna D Rao, Elizabeth Stierman, Aarushi Bhatnagar, Garima Gupta, Abdul Gaffar

**Affiliations:** a Public Health Foundation of India, New Delhi, India; b Johns Hopkins Bloomberg School of Public Health, Baltimore, MD, USA; c National Health Resource Center, Ministry of Health and Family Welfare, Government of India, New Delhi, India; d Alliance for Health Policy and Systems Research, World Health Organization, Geneva, Switzerland

## Abstract

**Background:**

Attracting physicians to rural areas has been a long-standing challenge in India. Government efforts to address the shortage of rural physicians include posting non-physician clinicians (NPCs) at primary health centers (PHCs) in select areas. Performance assessments of NPCs have typically focused on the technical quality of their care with little attention to the perspectives of patients. This study investigates patient views of physicians (Medical Officers) and NPCs in terms of patient satisfaction, perceived quality, and provider trust. NPCs include: Indian system of medicine physicians (AYUSH Medical Officers) and clinicians with 3 years of training, such as Rural Medical Assistants (RMAs). At PHCs without clinicians, paramedics provide clinical care, although they are not trained for this.

**Methods:**

PHCs in the state of Chhattisgarh were stratified by provider type: Medical Officer, AYUSH Medical Officer, RMA, or paramedic. PHCs were randomly sampled in each group. A total of 1,082 exiting patients were sampled from138 PHCs. Factor analysis was used to identify perceived quality domains. Multiple regression analysis was used to test for group differences.

**Results:**

Patients of Medical Officers and NPCs reported similar levels of satisfaction, trust, and perceived quality, with scores of 84% for Medical Officers, 80% for AYUSH Medical Officers, and 85% for RMAs. While there were no significant differences in these outcomes between these groups, scores for paramedical staff were significantly lower, at 73%.

**Conclusions:**

Physicians and NPCs performed similarly in terms of patient satisfaction, trust, and perceived quality. From a patient's perspective, this supports the use and scale up of NPCs in primary care settings in India. Leaving clinician posts vacant undermines public trust and quality perceptions of government health services.

## Abstract (precis)

Non-physician clinicians (NPCs), including both specially trained medical assistants and physicians trained in India systems of medicine, perform similarly to physicians in terms of patient satisfaction, trust, and perceived quality, thus supporting the use and scale up of NPCs in primary care.

## BACKGROUND

Clinical care providers with shorter duration of medical training than physicians provide primary health services in several developed and developing countries.^1^^–^^4^ In places where physicians are scarce, as in several countries in sub-Saharan Africa, non-physician clinicians (NPCs) have become the main providers of primary health care, and, in some instances, even provide specialist services.^1^,^5^^–^^8^ Evidence from both developed and developing countries suggests that NPCs can be effective primary care providers. In various settings they have been found to be as effective as physicians in managing conditions ranging from childhood illnesses, abortions, deliveries, emergency obstetric services, and cardiovascular conditions.^2^,^4^^–^^7^

Non-physician clinicians have been found to be as effective as physicians in managing conditions from childhood illnesses to emergency obstetric services and more.

### Patient Perspectives

Performance assessments of NPCs have typically focused on the technical quality of their care with little attention to the perspectives of patients. Yet it is important to understand patient perspectives, such as satisfaction with the NPC, the perceived quality of care they received, and patient trust in the NPC. These perspectives are positively correlated with a range of behaviors, such as willingness to seek care, choice of provider, adherence to prescribed treatment, and recommendation of provider.^8^^–^^14^

Patients' satisfaction reflects the extent to which expectations of service standards have been met, and their perceptions of quality can yield important information about different aspects of service quality.^15^ Patients' trust in a physician has been defined in several ways but generally refers to a patient's belief that the physician will act in the patient's best interest.^14^^,^^16^ Although satisfaction and trust are related, there are important distinctions. Trust carries an expectation of future behavior, while satisfaction is concerned with the past. And while satisfaction captures patient opinions of the physician, trust more directly refers to their relationship on the basis of patient perceptions about the physician's motivations.^16^^,^^17^ Trust has been conceptualized as having several domains covering physician competence, physician behavior, and global trust. The latter, which is a catch-all domain and employed in this study, captures aspects of trust in the different trust domains and beyond.^14^^,^^16^

Patients' satisfaction with and trust in their physicians affects their willingness to seek care and adhere to the prescribed treatment.

### Types of Non-Physician Clinicians

In India, several types of NPCs provide clinical care at primary health centers (PHCs). In many states, AYUSH physicians trained in Indian systems of medicine (Ayurveda, Yoga, Unani, Siddha, and Homeopathy) are posted at PHCs with the aim of bringing Indian systems of medicine into the mainstream. Often they are the sole clinician present and practice both allopathic and their own system of medicine. Clinicians with 3 years of training in allopathic medicine operate in 2 states—the state of Chhattisgarh has posted Rural Medical Assistants (RMAs) at PHCs, while in Assam state, similarly trained Rural Health Practitioners (RHPs) serve at sub-centers. More recently, the central health ministry proposed to introduce a nationwide 3-year clinician course, the Bachelor‘s of Rural Health Care (BRHC). There has, however, been much concern about introducing such cadres, with critics questioning their necessity, their ability to perform all the clinical functions of a physician, their acceptability, and claims that such a program reflects discrimination against rural people.^18^

### Patient Perceptions of Care

Despite the growing presence of NPCs in India, little is known about the quality of care they deliver and how patients view their services. An earlier study assessing the clinical competence of NPCs operating at PHCs in India found that RMAs are as competent as physicians in primary care settings, while AYUSH physicians received lower competence scores, and paramedics the lowest.^19^

The focus of this article is on patient views—their satisfaction, trust, and perceptions of quality of care—regarding the performance of physicians and NPCs. The study is set in the central Indian state of Chhattisgarh, where several types of clinicians serve at PHCs—physicians (Medical Officers), AYUSH Medical Officers, and clinicians with 3 years of allopathic training (RMAs). At many PHCs, paramedics (nurses and pharmacists) provide clinical services because no higher-level provider is available. However, they are neither trained nor expected to perform this job. In this study, AYUSH physicians and RMAs are considered NPCs. To the best of our knowledge, no study so far has examined patient perceptions of care provided by NPCs in a developing-country context.

### Training of Rural Medical Assistants

In response to the shortage of Medical Officers in rural areas, the state of Chhattisgarh started to train RMAs in 2001. They receive 3.5 years of training followed by 1 year of internship. In contrast, physicians possessing a MBBS receive 5.5 years of training, including a 1-year internship. The RMA curriculum is essentially a compressed MBBS (Bachelor of Medicine, Bachelor of Surgery) program.^20^ However, the RMA internship prepares the RMAs for rural service; RMAs spend 1 month at a sub-center, 3 months at a PHC, 4 months at a sub-district hospital, and 4 months at a district hospital where they are rotated through different departments. They receive a Diploma in Modern and Holistic Medicine on completing their training. RMAs can serve only at PHCs, and they perform all the clinical, public health, and administrative duties expected of a Medical Officer (except for post-mortems and medico-legal cases).

### Training of AYUSH Physicians

AYUSH physicians in this study are ayurvedic physicians. They possess a Bachelor of Ayurvedic Medicine & Surgery (BAMS) degree, which involves the same duration of training as the MBBS degree. They receive some exposure to allopathic medicine during training, and while in government service they are trained to manage conditions related to a range of national disease control programs such as malaria and tuberculosis. Available evidence indicates that it is common for AYUSH physicians to engage in “mixed practice” and prescribe allopathic medicines, although the legality of this is ambiguous.^21^^–^^23^

## DATA AND METHODS

### Ethics Statement

Ethical clearance for the study were received from the Public Health Foundation of India Institutional Review Committee and the World Health Organization's Research Ethics Review Committee. Consent was obtained following recommended guidelines.

### Sampling and Data Collection

Data for this study were collected between July and September 2009. The study uses a cross-sectional design in which PHCs in Chhattisgarh were first stratified by the primary clinical care provider (Medical Officer, AYUSH Medical Officer, RMA, paramedic) present. A random sample of PHCs was drawn from each stratum to select a representative sample. Patients were sampled from the selected PHCs regarding their perceptions of the care received from the clinical provider.

In the first stage, a complete listing of PHCs and the staff present was compiled based on information supplied by the Department of Health and Family Welfare, Chhattisgarh, and verified with district health system managers. The staffing pattern of PHCs allowed the 706 PHCs in Chhattisgarh to be classified into 6 groups according to the main clinical care provider present: Medical Officers regular (210) and contractual (123), AYUSH Medical Officers (169), RMAs (63), paramedics (53), and others (88). The paramedical category comprised pharmacists, nurses, and others. The “others” category included auxiliary nurse midwives, dressers, and other support staff. In the rare instance where there was more than one clinician present, typically a Medical Officer (allopathic) and an AYUSH Medical Officer, the PHC was assigned to the senior-ranking clinician's (for example, Medical Officer) group.

PHCs who could not be reached because of poor roads or poor security were excluded. Further, contractual Medical Officers were excluded because they are qualified similarly to their regular counterparts. PHCs in the “others” group were excluded because they do not provide clinical care. The reduced sampling frame comprised 456 PHCs and the relevant groups limited to regular Medical Officers (205), AYUSH Medical Officers (135), RMAs (63), and paramedics (53). Simple random sampling without replacement was used to select 40 PHCs in each of the 4 groups included in the study.

A convenience sample of 10 outpatients was selected as they exited the PHC. Only those patients visiting the PHC for the first time for their current illness, and only those who were patients of the main clinical provider, were eligible for interviews. Patients, or their caregivers in the case of children, were interviewed after taking informed consent.

A total of 1,082 patients were interviewed at 138 PHCs, achieving 86.3% of the target sample size of 160 PHCs. Across all 4 groups, at least 80% of the target sample size was achieved. The target sample size of PHCs was not achieved completely because 14 clinical providers could not be contacted during the first stage; during the second stage, 3 PHCs could not be reached because of poor roads or poor security, and at 5 PHCs no patients were available when the surveyors visited during clinic hours. At several PHCs, fewer than the quota of 10 patients visited on the day the survey team arrived, which also contributed to the patient sample falling short of the target.

### Patient Questionnaire

A structured questionnaire was used to collect information from patients on their socioeconomic background, satisfaction with services, and the quality of care they had just received. Patient satisfaction was measured by asking patients, “How satisfied are you with your visit to this health facility?” Possible responses were “satisfied,” “neither satisfied nor dissatisfied,” and “not satisfied.”

Patient perceptions of quality were measured by asking patients to rate their level of agreement with a series of statements on different aspects of the service they had just experienced (for example, “the doctor gave you adequate time”). Responses were recorded on a 5-point Likert scale ranging from “completely disagree” to “completely agree,” and a neutral point of “neither disagree nor agree.” Scale items used in this study were taken from an earlier 16-item scale with good validity and reliability that was developed by the authors for inpatients and outpatients at a range of health facilities in India.^24^ Two items related to the availability of toilets and drinking water were dropped because few outpatients experience these facilities during their visit. One item related to ease of obtaining drugs at the PHC was dropped because it added little new information. The final list contained 13 items.

Patient trust in the physician was measured using the single global trust item, “You trust the skills and abilities of the doctor.”^16^ Responses were recorded on a 5-point Likert scale.

Additional survey instruments were used to collect information on the background of the main clinical provider, the condition of the PHC, and characteristics of the village where the PHC is located.

### Statistical Methods

The response rate to the questionnaire was high. Of the sample of 1,082 patients interviewed, there were a total of 8 non-responses across the 13 perceived quality items (0.05%). Missing values were imputed using the mean of the individual's scores for the remaining non-missing items.

Principal component analysis was used to determine dimensions of patient-perceived quality based on the 13-item scale. The importance of a component was evaluated by examining both scree plots and the contribution of each component to total variance (≥5%). Maximum likelihood factor analysis with varimax rotation was then applied, with the principal component analysis results guiding the number of factors to be extracted. Items with substantial loadings (≥0.49) on a single factor were retained. This process was repeated until all items had substantial loadings.

Perceived quality scores for the 4 dimensions of quality and on the single trust item were calculated by averaging the item response values. Average scores ranged from 1 to 5, with 5 being the highest quality rating. Scores were standardized so that each score was expressed in terms of number of standard deviations from the sample mean. Internal consistency reliability of the perceived quality scale was measured using Cronbach's alpha. Since the focus of this paper is on the PHC clinician, only results relating to the clinical consultation are presented.

Of interest is the difference in average perceived quality and trust scores between clinical provider groups (Medical Officer, AYUSH Medical Officer, RMA, and paramedic). Because provider quality, and consequently perceptions of quality, can be influenced by individual and contextual factors, we control for patient, PHC, and village characteristics using multiple regression.^25^ In estimating group differences, the Medical Officer group was taken as the reference category. Because observations from interviews conducted at the same PHC will likely be correlated due to unobserved provider and facility effects, we applied robust clustering to the regression model with the PHC as the unit of clustering.

The independent variables included in the multiple regression model are detailed below. Patient characteristics included sex, age, household size, literacy, self-reported waiting time, and a “wealth index” constructed using principal component analysis of 11 items indicating ownership of selected household assets (for example, bicycle, cattle, radio).^26^ PHC clinician characteristics included age, sex, and number of years he or she had worked at the PHC level. Facility characteristics included a PHC Infrastructure Index constructed using principal component analysis of 15 items related to the facility's infrastructure, such as the availability of electricity, water, and specific rooms for drug storage, cold chain, and consultations. Other variables included a measure of remoteness of the PHC's location (distance from the PHC to nearest road) and how well it was supplied (number of drug stockouts in the past year). Village-level characteristics included whether the village was located in a tribal area, and a Village Development Index was constructed using principal component analysis of 7 items indicating the presence of a secondary school, senior secondary/high school, regular electricity supply, piped water, tube wells, regular bus service, and cell phone connectivity. Stepwise regression methods were used to arrive at the final regression model. Model fit and assumptions were checked using residual plots. The statistical package STATA 8.2 was used for all statistical analysis.^27^

## RESULTS

### Sample Characteristics

Across all provider categories, the sample of patients interviewed was similar in proportion of males to females (3:2), average age (27 years), and household size (6–7 members) (see Table 1). The age of patients ranged from under 1 to 81 years, with a similar age distribution across providers. Fever was the most common complaint in all groups (35%), followed by pain (15%), injury (8%), diarrhea (8%), and cough (6%). The literacy level among patients in the paramedical group (66% literate) was slightly lower than in other groups (71–74% literate). Patients visiting Medical Officers and RMAs had, on average, higher wealth index scores than the other 2 groups.

**Table 1.** Characteristics of the Sample

	**Medical Officer**	**AYUSH Medical Officer**	**Rural Medical Assistant**	**Paramedical Staff**	**All Providers**
**Patient Characteristics**					
Male^a^	153 (57%)	184 (62%)	168 (62%)	139 (57%)	644 (60%)
Age (years)^b^	27.4 (19.6)	22.0 (17.5)	29.8 (21.7)	28.3 (21.6)	26.7 (20.3)
Literate^a^	192 (71%)	218 (74%)	203 (74%)	160 (66%)	773 (71%)
Household size^b^	6.6 (3.2)	6.3 (2.6)	6.2 (3.2)	6.7 (3.3)	6.4 (3.1)
Wealth index^b^	0.24 (1.9)	−0.48 (1.6)	0.23 (1.8)	0.06 (1.7)	0.0 (1.8)
Waiting time >10 minutes^a^	60 (22%)	40 (14%)	28 (10%)	7 (2.9%)	135 (13%)
No. of Observations	269	296	273	244	1082
**Provider Characteristics**					
Male^a^	26 (81%)	33 (94%)	23 (64%)	29 (83%)	111 (80%)
Age^b^	41.8 (7.1)	34.9 (6.2)	26.3 (1.8)	33.2 (11.3)	33.8 (9.1)
Experience at PHC level (months)^a^	145 (83.6)	39 (20.9)	11 (1.8)	108 (130)	74 (93)
No. of Observations	32	35	36	35	138
**Facility Characteristics**					
No. of drug stockouts in past year^b^	1.8 (3.0)	1.5 (2.5)	1.4 (1.5)	1.8 (2.8)	1.6 (2.5)
PHC infrastructure index^b^	0.84 (2.2)	−0.35 (1.6)	0.16 (2.0)	−0.59 (1.8)	0.0 (2.0)
No. of Observations	32	35	36	35	138
**Village Characteristics**					
Tribal^a^	8 (25%)	26 (74%)	12 (33%)	9 (26%)	55 (40%)
Village development index^b^	0.50 (1.0)	−0.85 (1.9)	0.05 (1.0)	0.34 (1.2)	0.0 (1.4)
Distance from PHC to nearest road (km) b	1.8 (4.4)	2.1 (5.3)	12.1 (66.5)	2.4 (5.1)	4.7 (34.2)
No. of Observations	32	35	36	35	138

Abbreviation: PHC, primary health center.

a N (%)

b Mean (standard deviation)

Among providers, the RMA group had the highest proportion of female providers (36%). RMAs were also, on average, younger and had spent less time working at the PHC level. This is unsurprising given the recent creation of the RMA cadre in 2001.

Facilities where AYUSH Medical Officers and paramedical staff were stationed had lower infrastructure index scores, on average, than those of Medical Officers and RMAs. AYUSH Medical Officers more often worked in tribal areas and in villages with lower levels of development.

Patient satisfaction was high for Medical Officers (84%), AYUSH Medical Officers (80%), and rural medical assistants (85%) but lower for paramedics (73%).

### Patient Satisfaction

Overall, most patients (81%) reported being satisfied with their visit to the PHC (Table 2). Patient satisfaction in the Medical Officer (84%), AYUSH Medical Officer (80%), and RMA (85%) groups was high. However, the paramedical group reported a lower proportion of satisfied patients (73%).

**Table 2.** Patient Perceptions of Quality and General Satisfaction Scores

	**Medical Officer**	**AYUSH Medical Officer**	**Rural Medical Assistant**	**Paramedical Staff**	**Cronbach's Alpha**
Patient Satisfaction, N (%)^a^	224 (84%)	238 (80%)	231 (85%)	178 (73%)	
*Dimensions of Perceived Quality*					
*Medical Advice (3 items)*					0.74
Standardized score, mean (95% CI)	0.07 (−0.13, 0.26)	−0.11 (−0.25, 0.04)	0.17 (−0.04, 0.31)	−0.14 (−0.39, 0.11)	
Adjusted mean difference, mean (95% CI)^b^	Reference	−0.08 (−0.29, 0.14)	0.19 (−0.08, 0.45)	−0.12 (−0.40, 0.16)	
*Medical Consultation (4 items)*					0.79
Standardized score, mean (95% CI)	0.12 (−0.09, 0.33)	−0.06 (−0.23, 0.12)	0.19 (0.01, 0.36)	−0.27 (−0.50, −0.04)	
Adjusted mean difference, mean (95% CI)^b^	Reference	−0.06 (−0.26, 0.15)	0.12 (−0.12, 0.35)	−0.21 (−0.44, 0.01)	
*Staff Behavior (3 items)*					0.82
Standardized score, mean (95% CI)	−0.03 (−0.15, −0.09)	−0.08 (−0.23, 0.07)	0.09 (−0.08, 0.26)	0.03 (−0.17, 0.23)	
Adjusted mean difference, mean (95% CI)^b^	Reference	0.09 (−0.06, 0.24)	0.15 (−0.04, 0.33)	0.08 (−0.09, 0.25)	
*Facility Infrastructure (3 items)*					0.69
Standardized score, mean (95% CI)	0.06 (−0.12, 0.24)	−0.06 (−0.23, 0.11)	0.14 (−0.06, 0.35)	−0.15 (0.51, 0.21)	
Adjusted mean difference, mean (95% CI)^b^	Reference	0.10 (−0.14, 0.34)	0.23 (−0.06, 0.52)	−0.002 (−0.25, 0.24)	
Patient's Trust in Clinician's Skill and Ability					
Standardized score, mean (95% CI)	0.24 (0.10, 0.38)	−0.04 (−0.20, 0.12)	0.03 (−0.14, 0.19)	−0.24 (0.46, −0.02)	
Adjusted mean difference, mean (95% CI)^b^	Reference	−0.12 (−0.29, 0.05)	−0.08 (−0.27, 0.11)	−0.30 (−0.47, −0.14)	
Observations	269	296	273	244	
Number of facilities (clusters)	32	35	36	35	

a Five values for general patient satisfaction were missing; missing values were omitted from calculations.

b Results for AYUSH, Rural Medical Assistant, and paramedical are the difference in mean scores between their group and the Medical Officer group, after controlling for the sex, age, wealth, and literacy of the patient; the sex and age of the clinical provider; and facility infrastructure and location (tribal area and distance to nearest road).

### Perceived Quality

The full range of responses was observed on each scale item (Table 3), although responses were skewed towards higher values. Factor analysis results are shown in Table 2. Four components of perceived quality were identified: medical advice, medical consultation, staff behavior, and facility infrastructure. These 4 dimensions correspond to those in the source study for the scale items.^24^ All 4 dimensions had fairly good reliability with Cronbach alpha values above 69%. Perceived quality scores on the dimensions of medical advice (3.3) and medical consultation (4.0) were above average (maximum 5.0).

**Table 3.** Rotated Factor Loadings for Scale Items Measuring Patient Perceptions of Quality

**Scale Items**	**Factor 1 Medical Advice**	**Factor 2 Medical Consultation**	**Factor 3 Staff Behavior**	**Factor 4 Facility Infrastructure**
The doctor gave you complete information about your illness	**0.7639**	0.1559	0.1004	0.076
The doctor gave you complete information about your treatment	**0.7236**	0.1571	0.1518	0.1122
The doctor gave you advice about ways to avoid illness and stay healthy	**0.493**	0.2062	0.1809	0.1392
Staff of the health facility talked to you politely	0.1226	0.1797	**0.7163**	0.1055
Staff of the health facility were helpful	0.1567	0.195	**0.7398**	0.1342
Staff behavior was good	0.0797	0.2126	**0.7501**	0.1352
The doctor gave you adequate time	0.1711	**0.5901**	0.3463	0.0986
The doctor listened carefully to what you had to say	0.1172	**0.6832**	0.2631	0.0777
The doctor checked you properly	0.269	**0.6117**	0.1441	0.2124
The doctor was ready to answer all your questions	0.1703	**0.6462**	0.2189	0.0969
Cleanliness of the health facility was adequate	0.1205	0.0621	0.1663	**0.5349**
This health facility had all requisite amenities	0.107	0.1453	0.1366	**0.7675**
This health facility had all the drugs you needed	0.1072	0.1065	0.1984	**0.5522**

Items in boldface represent those that have high loadings on a particular factor. Items with high loadings were then used to interpret the factor and to construct the perceived quality sub-scales.

### Perceived Quality of Medical Advice

Overall, the average score for the medical advice dimension was 3.3 (maximum 5.0). The standardized scores for RMAs and Medical Officers were 0.17 and 0.07 standard deviations above the sample mean, respectively; for AYUSH Medical Officers, the scores were 0.11 and 0.14 standard deviations below the sample mean, respectively (Table 2). No significant differences (95% CI includes 0) were observed between Medical Officers and any of the other groups, both before and after adjusting for provider, patient, PHC, and area characteristics.

### Perceived Quality of Medical Consultation

The average score for the medical consultation dimension was 4.0 (maximum 5.0). Standardized scores were highest for RMAs (0.19) followed by Medical Officers (0.12), AYUSH Medical Officers (−0.06) and paramedics (−0.27) (Table 2). Significant differences were found only between the paramedical group and Medical Officers. After controlling for provider, patient, PHC, and area characteristics, no significant differences were observed between the Medical Officers and other groups. However, the 95% confidence interval for the adjusted mean difference between the Medical Officer and paramedical groups almost excludes 0.

### Patient Trust

Patient trust in PHC clinicians was high, with the average score being 4.2 (maximum 5.0). Standardized scores were highest for the Medical Officer group (0.24), followed by RMA (0.03), AYUSH Medical Officer (−0.04) and paramedical (−0.24) (Table 2). Only paramedical staff had significantly lower average adjusted standardized scores than Medical Officers (95% CI excludes 0).

## DISCUSSION

Performance assessments of non-physician clinicians have typically focused on the technical quality of their care and largely ignored the perspectives of patients. Interestingly, meta-analyses of studies on nurse-practitioners operating in developed countries have found that patient health outcomes were similar for nurses and doctors, but patient satisfaction was higher with nurse-led care.^2^^,^^28^^,^^29^ As mentioned above, an assessment of the clinical skills of RMAs and AYUSH Medical Officers in Chhattishgarh found that the clinical competency of Medical Officers was similar to that of RMAs, but significantly higher than that of AYUSH Medical Officers, for managing common conditions seen in primary care settings.^19^

### Patients Equally Satisfied with NPCs and Physicians

In this study, we examined patient views of different types of clinical care providers operating at PHCs in Chhattisgarh state. Patients were equally satisfied with Medical Officers, AYUSH Medical Officers, and RMAs. Further, patients reported similar levels of trust in the ability of these clinicians, and similar ratings of quality, in terms of medical consultation and medical advice. No statistically significant differences were found between Medical Officers and RMAs, nor between Medical Officers and AYUSH Medical Officers, on any of these indicators. In contrast, on all these indicators, paramedics received significantly lower scores than Medical Officers (except for perceived quality of medical advice). These findings indicate that, from the perspective of patients, AYUSH Medical Officers and RMAs appear to be as acceptable as Medical Officers. This provides support to the policy of deploying NPCs at PHCs in India.

Non-physician clinicians (NPCs) appear to be as acceptable to patients as Medical Officers, providing support to the policy of deploying NPCs at India‘s understaffed primary health centers.

### Poor Care and the Erosion of Trust

Paramedics consistently received the lowest patient evaluations, whether it was for satisfaction with services, trust in their abilities, or ratings of the quality of the consultation. This is expected since the paramedics in the sample (31 pharmacists, 2 nurses, and 2 other paramedics) were not trained to provide clinical care but did so because no qualified clinician was present. It is important to recognize that in many other countries, nurses with special training (for example, nurse clinicians, nurse-practitioners) do provide quality clinical care, including prescribing medications.^1^^,^^2^^,^^28^^,^^29^ However, no such cadre of nurses currently exists in India. Neither are pharmacists trained to diagnose and treat patients, and the high number of pharmacists who are providing clinical care is worrying. This highlights the danger of leaving clinician posts vacant at health facilities. Moreover, the presence of poorly qualified providers undermines public trust and perceptions of quality in the services offered and in government health services more generally.

### Other Factors to Consider

The levels of patient satisfaction with services at the local PHC reported in this study were similar to those reported for government health facilities in large household surveys in India.^30^ All exit interviews were conducted at PHCs, and patients referred to the Medical Officers, AYUSH Medical Officers, and RMAs as “doctor,” indicating that these cadres' identities were not differentiated by patients (or in the questionnaire). Paramedics (pharmacists and nurses), however, were known by their cadre identities. The case mix and patient characteristics, which influence user views on services, can systematically vary across groups, given that geographic location or the type of provider present might influence care-seeking behavior. When we investigated this, we found that the distribution of presenting symptoms was similar across groups, suggesting that the case mix might also be similar between groups. Further, in the analysis we attempted to minimize this factor by examining group differences after controlling for patient and other characteristics. Because our analysis was based on a convenience sample of users (patients at health facilities), findings from this study cannot be generalized to the larger population. Non-users are likely to have different perceptions of PHCs and the providers present there compared with users. For instance, we would expect non-users to have lower trust or satisfaction with the local PHC. Finally, issues of absenteeism continue to be a problem, even with NPCs.

### Severe Staffing Shortages at Primary Health Centers

In several countries, NPCs play an important role in delivering basic health services. To increase the presence of clinicians in rural communities, several states in India have staffed PHCs with NPCs such as AYUSH Medical Officers or clinicians with shorter duration of clinical training such as RMAs. At the time of this study, only 47% of the PHCs in Chhattisgarh had a physician present, indicating the severe shortage of physicians in rural health centers. In 32% of the PHCs, which were mostly located in remote and tribal areas, the presence of either RMAs or AYUSH Medical Officers enabled these health centers to continue providing clinical care. Our findings indicate that patients had similar levels of satisfaction, trust, and perceived quality with Medical Officers and NPCs.

### Non-Physician Clinicians Are Part of the Solution

Successful primary health care is built on the trust and rapport between clinicians and the communities they serve. As India attempts to achieve health care for all, reducing the shortage of qualified clinicians in underserved areas needs to become a priority. NPCs, whether they are clinicians with 3 years of training, nurse-practitioners, or AYUSH physicians, can be an important part of the solution, as long as they possess a standard level of clinical competence, and their patients are satisfied with—and trust—the care they receive.
